# Vesicle-independent extracellular release of a proinflammatory outer membrane lipoprotein in free-soluble form

**DOI:** 10.1186/1471-2180-8-18

**Published:** 2008-01-28

**Authors:** Maribasappa Karched, Riikka Ihalin, Kjell Eneslätt, Deyu Zhong, Jan Oscarsson, Sun N Wai, Casey Chen, Sirkka E Asikainen

**Affiliations:** 1 Oral Microbiology, Department of Odontology, Umeå University, SE-90187 Umeå, Sweden; 2 Department of Molecular Biology, Umeå University, SE-90187 Umeå, Sweden; 3 Primary Oral Health Care, USC School of Dentistry, University of Southern California, 925 W. 34^th ^Street, Los Angeles CA 90089-0641, USA; 4 Department of Biochemistry and Food Chemistry, University of Turku, Turku FIN-20520, Finland; 5 Department of Dentistry, Medical School Hospital of Qingdao University, Qingdao, People's Republic of China

## Abstract

**Background:**

*Aggregatibacter actinomycetemcomitans *is an oral bacterium associated with aggressively progressing periodontitis. Extracellular release of bacterial outer membrane proteins has been suggested to mainly occur via outer membrane vesicles. This study investigated the presence and conservation of peptidoglycan-associated lipoprotein (AaPAL) among *A. actinomycetemcomitans *strains, the immunostimulatory effect of AaPAL, and whether live cells release this structural outer membrane lipoprotein in free-soluble form independent of vesicles.

**Results:**

The *pal *locus and its gene product were confirmed in clinical *A. actinomycetemcomitans *strains by PCR-restriction fragment length polymorphism and immunoblotting. Culturing under different growth conditions revealed no apparent requirement for the AaPAL expression. Inactivation of *pal *in a wild-type strain (D7S) and in its spontaneous laboratory variant (D7SS) resulted in pleiotropic cellular effects. In a cell culture insert model (filter pore size 0.02 μm), AaPAL was detected from filtrates when strains D7S and D7SS were incubated in serum or broth in the inserts. Electron microscopy showed that *A. actinomycetemcomitans *vesicles (0.05–0.2 μm) were larger than the filter pores and that there were no vesicles in the filtrates. The filtrates were immunoblot negative for a cytoplasmic marker, cyclic AMP (cAMP) receptor protein. An ex vivo model indicated cytokine production from human whole blood stimulated by AaPAL.

**Conclusion:**

Free-soluble AaPAL can be extracellularly released in a process independent of vesicles.

## Background

*Aggregatibacter actinomycetemcomitans *[1] is a small Gram-negative rod implicated in aggressive forms of periodontitis [2]. We recently identified and characterized in *A. actinomycetemcomitans *a 17-kDa peptidoglycan-associated lipoprotein (PAL) [3,4], which is a widely conserved outer membrane lipoprotein (OMLP) among Gram-negative bacteria [5]. The PALs of several pathogens, such as *Haemophilus influenzae*, *Escherichia coli *[6-9], *Legionella pneumophila, Bordetella pertussis*, and *Campylobacter jejuni *[10-12] are proinflammatory and/or strongly immunogenic and can contribute to serum resistance of the bacterium [13]. PAL may be an important bacterial mediator in sepsis caused by Gram-negative bacteria [7] and is crucial for full expression of virulence of *Haemophilus ducreyi *[14].

Extracellular release of bacterial virulence factors is a principal mechanism of bacterial pathogenicity [15]. Different strategies of release include specialized secretory systems [15-17], outer membrane vesicles [18,19], and autolysis [20]. Release of outer membrane vesicles is regarded as the primary mechanism for delivering structural surface components, such as outer membrane proteins (OMP) and lipopolysaccharide (LPS), into the extracellular milieu. Most previous studies on the released bacterial outer membrane components, however, have focused on detecting and isolating them from serum or culture supernatants, or on testing their biological activity [21-25]. Less attention has been directed to elucidating whether these components in the test material were derived from lysed cells, outer membrane vesicles, and/or were released from live bacteria in free-soluble form. Importantly, the bacterial cell components that are released in free-soluble form may be biologically more active than their membrane-bound counterparts associated with intact bacterial cells or outer membrane vesicles [26].

The present study was designed to investigate the presence and conservation of PAL among clinical *A. actinomycetemcomitans *strains and whether live *A. actinomycetemcomitans *cells release PAL (AaPAL) in free-soluble form. To accomplish these objectives, inserts with 0.02-μm pore-size filter were used to separate bacterial cells from cell culture wells containing serum or broth. We present compelling evidence for the release of AaPAL in free-soluble form by demonstrating presence of AaPAL in the filtrates obtained after incubation, by monitoring bacterial viability in the insert, and by checking for the absence of cell lysis and outer membrane vesicles in the filtrate. Proinflammatory effect of AaPAL was shown in an ex vivo model using human whole blood.

## Results

### The *pal *gene was conserved and the protein expressed in clonally diverse *A. actinomycetemcomitans *strains

**Figure 1:**
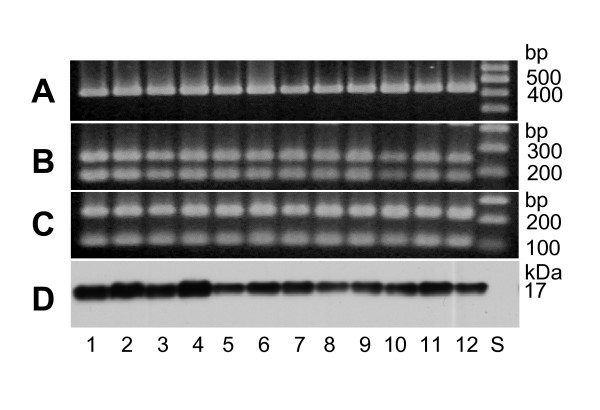
PCR-RFLP of *pal *and detection of its gene product, PAL, from clonally diverse *A. actinomycetemcomitans *strains. Panel A: Agarose gel electrophoresis of the PCR products shows amplicons with the expected size of *pal *(425 bp) for each strain. Panels B and C: Agarose gel electrophoresis of the purified PCR amplicons digested with *Dde*I and *Bsp*MI, separately. Panel D: immunoblot analysis of the *A. actinomycetemcomitans *whole cell protein preparations using anti-AaPAL peptide antiserum shows the expected 17-kDa signal for each strain. Lanes 1 through 12 strain identification (serotype; genotype): ATCC 29523 (a; 1), SA5002 (a; 1), ATCC 43718 (b; 2), SA5003 (b; 8), ATCC 33384 (c; 3), SA5005 (c; 3), SA5001 (d; 5), SA5007 (d; 22), SA5008 (e; 6), SA5011 (e; 20), CU1000R (f; nd*), SA5022 (f; 19) and standards (S). *nd: not determined.

A total of 33 *A. actinomycetemcomitans *strains were analyzed by PCR and immunoblotting. The results from 12 strains representing 6 serotypes and 9 genotypes are shown (Fig. 1). The genomic DNA and the whole cell protein preparation of these strains each produced the expected 425-bp amplicon in PCR and the 17-kDa immunoblot signal with anti-AaPAL peptide antiserum, respectively.

The RFLP analysis with two enzymes, *Dde*I and *Bsp*MI, cutting once within the *pal *sequence [4] showed that the restriction patterns were similar among all test strains (Fig. 1). The *Dde*I-digested fragments were 175 and 250 bp, as predicted, whereas the *Bsp*MI-digested fragments were 100 and 225 bp, which were not the predicted sizes of 324 and 101 bp, respectively. The reason seems to be a recognition site for *Bsp*MI on the opposite strand approximately 100 bp away from the reverse primer binding sequence.

### AaPAL was expressed in various culture conditions

**Figure 2:**
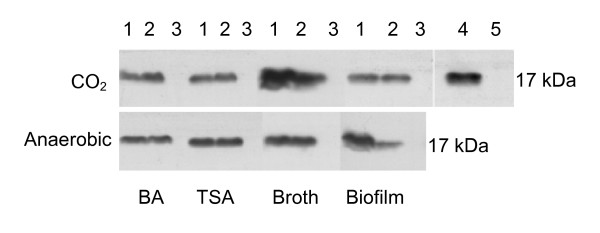
AaPAL expression in different growth conditions of *A. actinomycetemcomitans*. Strains D7S (wild-type, lane 1), D7SS (spontaneous smooth-colony variant of D7S, lane 2), and D7SS-p (*pal*-deficient mutant of D7SS, lane 3) were cultured in various nutritional and atmospheric conditions (for details, see Materials and Methods). Bacterial whole cell protein preparations (10 μg/well) were subjected to immunoblot analysis with anti-AaPAL peptide antiserum. The OMP preparations from the strains D7SS (lane 4) and D7SS-p (lane 5) were used as a positive and a negative control, respectively. CO_2_: CO_2_-enriched air (5%); BA: blood agar; TSA: trypticase soy agar.

To determine the environmental requirements for AaPAL expression, we cultured *A. actinomycetemcomitans *strains in different nutritional and atmospheric conditions. Besides conventional cultures, strains were also grown as biofilm [27]. The immunoblot analyses of the whole cell protein preparations of strains D7S and D7SS, but not D7SS-p, revealed 17-kDa signals, regardless of the culture conditions (Fig. 2). Furthermore, the 17-kDa band appeared stronger when strains D7S and D7SS had been grown in broth incubated in CO_2_-supplemented (5%) air than when grown in other conditions. A particularly strong band was also seen for strain D7S grown as biofilm under anaerobic incubation.

### Phenotype of the *pal*-deficient mutants

The strain D7SS-p was constructed to be used as a negative control in the experiments. The strain D7S-p was also constructed to serve as a negative control, but particularly in the experiments aimed to demonstrate with a clinical strain, D7S, that AaPAL release was not restricted to its spontaneous laboratory variant, D7SS.

**Figure 3:**
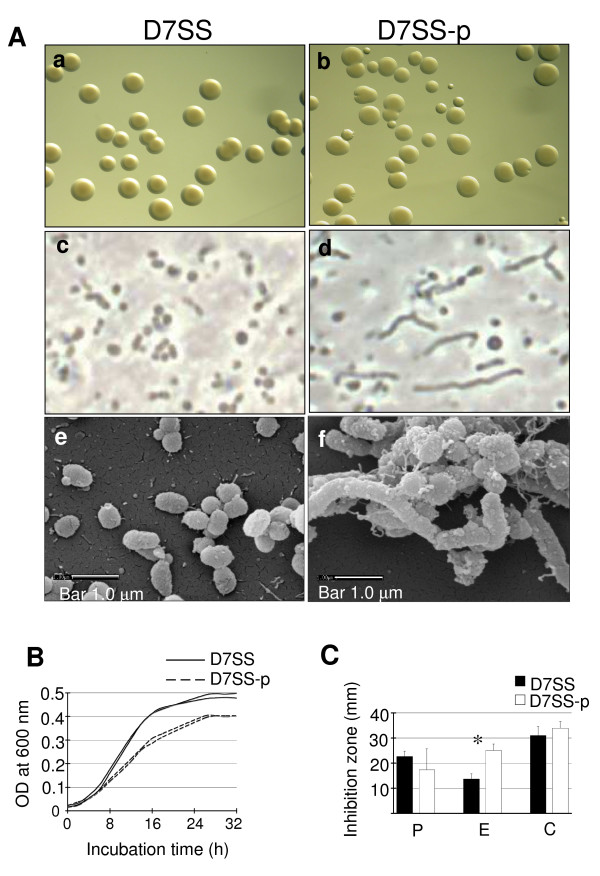
Role of AaPAL in cell physiology and membrane integrity. Panel A: Colony density and morphology (a, b) and the cell morphology of *A. actinomycetemcomitans *strains D7SS and D7SS-p grown on TSA as examined by phase contrast microscopy (c, d) and scanning electron microscopy (e, f). Panel B: Growth rates of D7SS and D7SS-p in TSB measured by turbidimetry (OD_600_). The results are shown from two separate experiments. Panel C: Antibiotic susceptibility comparison between the strains D7SS and D7SS-p. The results are means (SD) from three independent experiments (*P < 0.05). P, penicillin; E, erythromycin; C, cefotaxime.

Comparison between the phenotypes of the mutants and their parents (data for D7SS and D7SS-p are shown in Fig. 3) showed no distinct differences in the colony sizes or densities when standardized numbers of cells (1 × 10^3^) were cultured on plates. The mutants, however, exhibited filamentous cell morphology when observed by wet mounting (phase contrast microscopy) and SEM. The altered cell morphology was more pronounced when bacteria were cultured in nutritionally poor (TSA) rather than in rich (supplemented blood agar) medium (data not shown). As judged from the SEM analysis, there was enhanced vesicle production by the strain D7SS-p relative to D7SS (Fig. 3Ae and 3f). Furthermore, the growth rate of the mutant appeared inferior to the parent (Fig. 3B). The antibiotic susceptibility comparison between the strains showed that for the mutant, the inhibition zone was larger (P < 0.05) for erythromycin but not for cefotaxime or penicillin (Fig. 3C).

### AaPAL was detected in serum and broth filtrates

**Figure 4:**
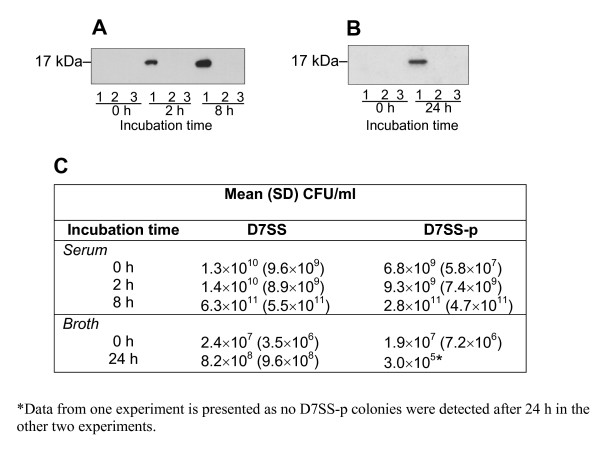
Immunoblot detection of PAL release from *A. actinomycetemcomitans *cells exposed to heat-inactivated FCS or cultured in broth. The samples were obtained from cell-culture wells outside the inserts, where the bacteria [D7SS (lane 1) and D7SS-p (lane 2)] or No bacteria (lane 3) were incubated in serum for 2 h and 8 h (Panel A) or cultured in broth for 24 h (Panel B). AaPAL was detected by immunoblot using anti-AaPAL peptide antiserum. Samples for bacterial culture and enumeration were taken from the inserts containing serum or broth (Panel C) at the same time points as the samples for the immunoblots in Panels A and B. The results show means (SD) from three independent experiments.

To elucidate how AaPAL could be presented to the host during *A. actinomycetemcomitans *infection, we studied whether *A. actinomycetemcomitans *cells released AaPAL into extracellular space in free-soluble form. For this purpose, we designed an in vitro model, where standardized numbers of bacterial cells were exposed for up to 8 h to heat-inactivated FCS (Fig. 4A). To explore the possibility of AaPAL release during *A. actinomycetemcomitans *growth, another similar experiment using D7SS and D7SS-p was performed using culture broth and a longer incubation time (24 h) (Fig. 4B). Immunoblot analysis of the filtrates outside the inserts containing strain D7SS or D7S revealed an accumulation of AaPAL over time (data for D7SS and D7SS-p are shown in Fig. 4). No AaPAL was detected in the above experiments at any time point in the filtrate when either D7SS-p or D7S-p or No bacteria were incubated in the insert. In all of the above described serum and broth experiments, OMP preparations of strains D7SS and D7SS-p were used as positive and negative controls, respectively, in the immunoblot assays (data not shown).

Densitometric quantification indicated that for strain D7SS incubated in serum, AaPAL concentration after the first 2 h was 637 ng/ml and at 8 h was 932 ng/ml (a 1.5-fold increase from 2 h). Similarly, for strain D7S incubated in serum, AaPAL concentration after the first 2 h was 437 ng/ml and at 8 h was 750 ng/ml (a 1.7-fold increase from 2 h). For strain D7SS in broth, AaPAL concentration at 24 h was 194 ng/ml (for strain D7S, only serum incubation was performed).

To ensure the viability of the bacteria during the AaPAL release experiments described above, we employed bacterial viable counts for samples taken from serum and broth inside the inserts at the same time points as samples that were taken from filtrates for the immunoblot analyses (data for D7SS and D7SS-p are presented in Fig. 4C). In serum, the mean CFU (per ml) counts of D7SS were 1.1-fold higher at 2 h than at 0 h and 46.0-fold higher at 8 h than at 2 h. Similarly, the mean CFU counts of D7SS-p were 1.3-fold higher at 2 h than at 0 h and 30.0-fold higher at 8 h than at 2 h.

In broth, the mean CFU counts of D7SS were 34.3-fold higher at 24-h than at 0 h. In contrast, the CFU counts of D7SS-p were 62.2-fold lower at 24 h than at 0 h (Fig. 4C).

Since the AaPAL release experiment was performed for strains D7S and D7S-p in serum only, the viable count data are given here: The mean CFU counts of D7S were at 0 h 6.2 × 10^8^, at 2 h 8.7 × 10^8 ^(a 1.4-fold increase), and at 8 h 1.16 × 10^10 ^(a 13.3-fold increase from 2 h). The respective mean CFU counts for D7S-p were at 0 h 6.1 × 10^8^, at 2 h 9.5 × 10^8 ^(a 1.5-fold increase), and at 8 h 1.4 × 10^10 ^(a 15.0-fold increase from 2 h).

No bacterial growth was found in the samples from the serum or broth filtrates at any time point, ensuring that bacteria growing within the inserts had not passed through the filter and/or that the filtrates had not been contaminated.

**Figure 5:**
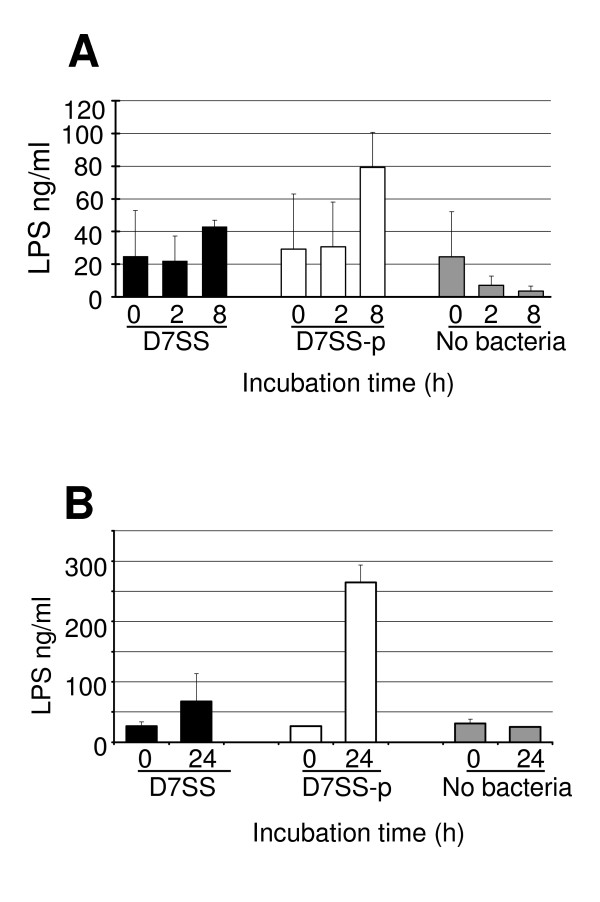
LPS released by *A. actinomycetemcomitans *cells using a cell culture insert model. Filtrates from serum incubation at 0, 2, and 8 h (Panel A) and broth culture at 0 and 24 h (Panel B) were subjected to quantification of LPS by Limulus assay. The results are means (SD) from two independent experiments.

For D7SS incubated in serum, although the mean LPS concentrations of the filtrates decreased 1.1-fold from 0 h to 2 h, there was an increase of 1.7-fold from 2 h to 8 h. The mean LPS concentrations for strain D7SS-p incubated in serum increased 1.0-fold from 0 h to 2 h and 2.6-fold from 2 h to 8 h (Fig. 5). During broth culture, the mean LPS concentrations for D7SS and D7SS-p increased 2.5- and 9.8-fold, respectively, from 0 h to 24 h. However, there were no statistically significant differences in the amounts of LPS in the serum or broth filtrates between the two strains at any time point or in regard to the No bacteria control between the different time points. At the 0 time point, the LPS concentrations of D7SS and D7SS-p in both serum and broth filtrates were almost equal to those of the respective No bacteria control samples (Fig. 5).

We also tested for the release of additional *A. actinomycetemcomitans *components, apart from PAL and LPS, in the filtrates. Immunoblot analysis of the filtrates after an 8-h serum incubation of D7SS, D7SS-p, and No bacteria was carried out by using an antiserum raised against whole cell antigen of *A. actinomycetemcomitans *serotype a (both D7SS and D7SS-p are of serotype a). Several immunoreactive bands of unknown identity but representing apparent molecular weights ≈ 10–120 kDa were detected in the filtrates from both D7SS and D7SS-p, but not in the No bacteria controls (data not shown).

### Extracellular release of AaPAL was independent of cell lysis

**Figure 6:**
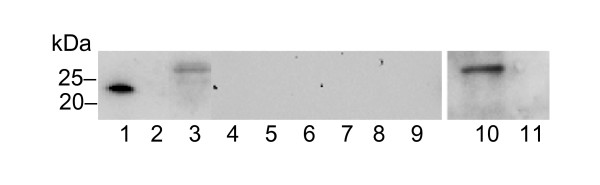
Immunoblot analysis of the filtrates for the detection of a cytoplasmic marker, cAMP receptor protein. The whole cell protein preparations of *V. cholerae crp*^+ ^strain (lane 1), *V. cholerae crp*^- ^strain (lane 2) and *A. actinomycetemcomitans *D7SS (lane 3) were used as controls in the immunoblot with antibodies against *V. cholerae *cAMP receptor protein. Serum filtrates (8 h): D7SS (lane 4), D7SS-p (lane 5) and No bacteria control (lane 6). Broth filtrates (24 h): D7SS (lane 7), D7SS-p (lane 8) and No bacteria control (lane 9). Detection of cAMP receptor protein from precipitated filtrates of lysed (lane 10) and unlysed D7SS cells (lane 11) incubated in the inserts containing broth for 8 h.

To exclude the possibility that the *A. actinomycetemcomitans *cells had lysed in the inserts, the filtrates in which AaPAL was detected were also tested by immunoblotting using specific antibodies against a cytoplasmic marker, cAMP receptor protein. Anti-*Vibrio cholerae *cAMP receptor protein antibodies were chosen since the protein is 79% identical and 94% similar to *A. actinomycetemcomitans *cAMP receptor protein. Whole cell protein preparation of *V. cholerae *(*crp*^+^) reacted at an expected 23 kDa size, whereas *A. actinomycetemcomitans *whole cell protein preparation showed an immunoreactive band at ≈ 27 kDa (Fig. 6). This molecular weight was higher than the weight deduced from its amino acid sequence, 24.2 kDa (Oralgen; Oral Pathogen Sequence Database).

Our findings demonstrate that cAMP receptor protein could not be detected in any of the filtrate samples obtained from the cell-culture insert model (Fig. 6), not even when the filtrates were concentrated with trichloroacetic acid precipitation (data not shown).

To show that soluble cAMP receptor protein could be detected in filtrates from lysed bacteria, deliberately broken (See Methods) *A. actinomycetemcomitans *cells were incubated in cell culture inserts containing broth for 8 h as described above. As shown in Fig. 6, filtrates from lysed but not from unlysed D7SS cells contained detectable amounts of cAMP receptor protein.

### AaPAL was associated with *A. actinomycetemcomitans *outer membrane vesicles

**Figure 7:**
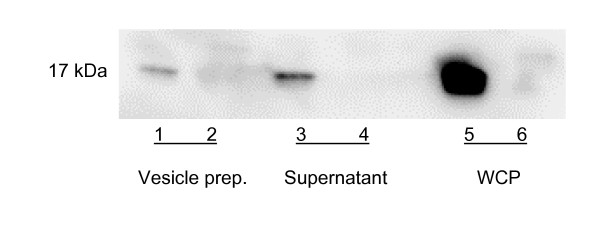
Immunoblot analysis of the vesicle preparations from *A. actinomycetemcomitans *strains D7SS and D7SS-p. The samples (5 μg protein each) were subjected to immunoblot analysis using anti-AaPAL peptide antiserum (1:500). The chemiluminescence signal was captured using a CCD camera integrated in ChemiDoc™ XRS gel documentation system (Bio-Rad). The images captured by the camera were acquired and imported to the attached computer using QuantityOne^® ^software (Bio-Rad). Lanes: D7SS (lanes 1, 3, 5) and D7SS-p (lanes 2, 4, 6). WCP; whole cell protein.

To investigate whether AaPAL could be detected in association with the vesicles, the vesicle preparations were analyzed using immunoblotting. A weak 17-kDa signal was detected in D7SS but not D7SS-p vesicles (Fig. 7), whereas the vesicle preparation supernatants obtained after ultracentrifugation (containing soluble bacterial components), showed a strong AaPAL signal for strain D7SS (D7SS-p was negative) (Fig. 7).

### Extracellular release of AaPAL was independent of outer membrane vesicles

**Figure 8:**
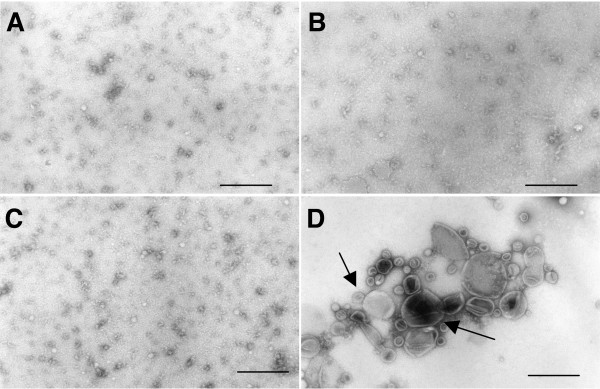
Electron micrographs of the vesicle preparations from filtrates. Filtrates from experiments studying in vitro AaPAL release into serum were subjected to vesicle preparation and subsequent electron microscopy. The filtrates were: *A. actinomycetemcomitans *D7SS at 8 h (Panel A), D7SS-p at 8 h (Panel B), No bacteria control at 2 h (Panel C). The vesicle preparation from *A. actinomycetemcomitans *D7SS whole cells (positive control) shows vesicles of different sizes indicated by arrows (Panel D). Bars; 0.2 μm.

Since AaPAL was detected in vesicle preparations from *A. actinomycetemcomitans *cultures (Fig. 7), we wanted to exclude the possibility that vesicles were the source of AaPAL in the filtrates. Electron microscopic examination of the vesicle preparations of the filtrates demonstrated large amounts of serum protein aggregates in all filtrates (Fig. 8). However, no vesicles were found in any of the filtrates at any time point. Data from time point 8 h are shown in Fig. 8. Vesicle preparations from D7SS whole cell suspension, used as a positive control, revealed vesicles of an average diameter 0.05–0.2 μm (Fig. 8).

### AaPAL induced cytokines in an ex vivo model

**Figure 9:**
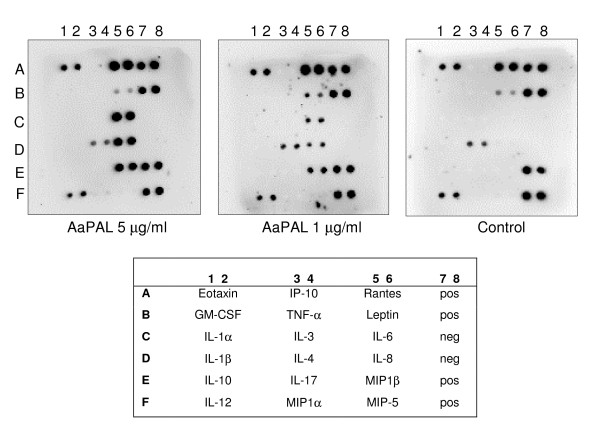
Cytokine induction of human whole blood by AaPAL. A cytokine antibody array was used to detect cytokines produced by human whole blood after stimulation with purified AaPAL (5 and 1 μg/ml of blood) for 8 h. Storage buffer of AaPAL served as the negative control. Abbreviations: pos = positive, neg = negative.

The effect of AaPAL on the cytokine milieu in human whole blood was profiled by using a cytokine antibody array and a preparation of affinity purified AaPAL, containing less than 5 pg LPS per μg of AaPAL [3]. Production of interleukins IL-6 and IL-8, and a macrophage inflammatory protein, MIP-1β, was demonstrated by enhanced signals (Fig. 9). Based on visual analysis the effects appeared to be concentration-dependent.

## Discussion

The present study demonstrates that *A. actinomycetemcomitans *releases AaPAL, a structural OMLP, in free-soluble form. The primary route for outer membrane components of bacteria, such as LPS and various proteins including porins [18,19,28,29] to the extracellular space is through the release of outer membrane vesicles. Although not widely studied, there are a few reports on the release of OMPs, such as OmpA, PAL, and major lipoprotein (Braun's lipoprotein), either in soluble form [21,25,30] or in the form of complexes [23,31]. In those studies, however, the methods were not designed to exclude the presence of vesicles in the samples [25,31,32] or bacterial death or lysis [21,25,30-32]. Our study made an attempt to control those factors and showed that AaPAL is released independent of vesicles. Release of free-soluble bacterial components, such as shown here for a lipoprotein, would be of biological significance, since these components can cross anatomical barriers impermeable to whole bacteria or even vesicles [33-35]. In addition, there is evidence to suggest that in free form bacterial components convey higher biological activity than when they are membrane-bound in intact bacteria or in vesicles [26]. Regardless, the results of the present study accentuate a less studied strategy by which oral bacteria, like bacteria in other localized infections, may express extended pathogenicity.

Our experimental model comprised a cell culture insert. Based on the previous literature on *A. actinomycetemcomitans *outer membrane vesicles [36], we chose a membrane filter with a pore size (0.02 μm) that would not allow cells or vesicles to pass through. An electron microscopic examination verified that the vesicle size for the present *A. actinomycetemcomitans *strains indeed was larger than the pores in the chosen filter. This renders unlikely the possibility that vesicles would have passed through the filter, which was further confirmed by the absence of vesicles in the filtrates. No literature was found about release of material from intact vesicles. Instead, vesicles are known to be considerably stable structures [37,38] and therefore, the possibility that lysed vesicles would have been the source of free-soluble AaPAL may be low.

Yet another possibility was that *A. actinomycetemcomitans *cells had died and lysed in the inserts, so we continuously monitored the viability of the bacteria grown in the inserts. The fact the total bacterial numbers increased over time shows that viability was maintained in the experiments. However, despite the increased total numbers, lysis of dying bacteria in the growing *A. actinomycetemcomitans *population may have presented a source of AaPAL in the filtrate. Therefore, we, like others [39,40], excluded the possibility of bacterial lysis by confirming the absence of a cytoplasmic marker in the same filtrate samples from which AaPAL was found.

Thus, neither vesicles passing through the filters nor cell death/lysis seemed to be the source of AaPAL in the filtrate samples. Further studies are needed to clarify the mechanism by which free-soluble AaPAL is released into extracellular space and how the release is regulated. Currently, it would be tempting to speculate that the mechanism involves activation of outer membrane phospholipase A [41-43]. By degrading phospholipids of the outer membrane, phospholipase A could liberate AaPAL along with other components of the outer membrane into the extracellular milieu. For other species, release of cell components, such as proteins, LPS and phospholipids has been reported following activation of phospholipase A, without an effect on growth or cell morphology [44]. Due to its strict regulation, phospholipase A activity does not lead to bacterial cell lysis [41,45].

For the biological activity of the released free-soluble AaPAL it is crucial that it retains the lipid domain, because lipoprotein-mediated cytokine induction is solely attributed to the lipid domain [46]. Our results support the retention of the lipid part of AaPAL, since the immunoblot detection from the filtrate demonstrated a band of the same MW (≈ 17 kDa) as that of purified AaPAL (without lipid domain, the amino acid sequence-based theoretical MW of AaPAL is ≈ 14 kDa). Therefore, the extracellularly released AaPAL seems to be neither proteolytically cleaved nor missing its lipid domain. The activity of free-soluble AaPAL may also be related to its association with other bacterial components. Our present study did not provide data to suggest an association but demonstrated co-release of AaPAL with LPS and other unidentified *A. actinomycetemcomitans *components. However, an association is possible, since previous studies have shown tight association between LPS and released *E. coli *PAL [8], as well as between extracellularly released OmpA, major lipoprotein, PAL and LPS [23,31].

Since in this study we were unable to obtain the extracellularly released AaPAL in pure form and therefore did not know whether or not it was complexed with other bacterial components, we chose to study the immunostimulatory effect of purified AaPAL in an ex vivo model. Our results showed a dose-dependent enhancement in the production of IL-6, IL-8, and MIP-1β after an 8-h stimulation, using purified AaPAL. A search of the literature revealed no previous studies of cytokine production from whole blood by using AaPAL, or other lipoproteins or OMPs of this species. The only study on other species also reported an increased IL-6 production from blood after incubation with *E. coli *PAL [8]. In general, IL-6 and IL-8 are among the major cytokines produced by whole blood after stimulation with bacterial components [47-52]. IL-6 has a wide range of biological activities, among them proinflammatory properties and a role as the main stimulus for acute phase protein synthesis and release from liver in infection [53]. One of the two chemokines induced by AaPAL in our study, IL-8, a prototypic CXC-chemokine and classically known as a neutrophil chemoattractant, mediates monocyte trafficking and functions as a macrophage stimulator [54]. The other chemokine, MIP-1β, a CC-subfamily chemokine, is a potent chemoattractant for monocytes and T cells [55]. Thus, even though we currently do not know the bioactivity of released free-soluble AaPAL, in purified form AaPAL has proinflammatory and prochemotactic properties that can lead to stimulation and differentiation of various cell types and further production of cytokines.

Our consistent results from RFLP of the *A. actinomycetemcomitans pal *gene and the immunoblot detection of the gene product indicate that the gene is conserved and the protein ubiquitously expressed in clonally diverse *A. actinomycetemcomitans *strains. This is in agreement with the intraspecies conservation of *pal *in other *Pasteurellaceae *members, such as *Haemophilus influenzae *[56]. The intraspecies conservation of AaPAL suggests that its bioactivity is comparable among different *A. actinomycetemcomitans *strains. The fact that AaPAL was expressed in a variety of different nutritional and atmospheric conditions may underscore its importance for the normal function of the bacterium. In addition, AaPAL was expressed during the exposure to serum and culture in anaerobic atmosphere. Since these conditions bear resemblance to those in pathologically deepened periodontal pockets, the results suggest that AaPAL is also expressed in vivo.

We included the *pal*-deficient mutant strains of *A. actinomycetemcomitans *primarily to corroborate our results from the immunoblot and other experiments. Comparisons of the phenotypic characteristics of the mutant and parent may, however, provide preliminary information about the role and function of AaPAL in *A. actinomycetemcomitans *cell physiology. We found that the mutants had decreased growth rate and filamentous cell morphology, differing partly from the *pal*-deficient mutant of *H. ducreyi*, which showed unchanged growth rate [14]. Although there were limitations in the present methods for estimating bacterial numbers, our results are consistent with previous reports demonstrating reduced growth rate of bacteria with mutations in *tol-pal *genes [13,57,58]. Moreover, the antimicrobial susceptibility of the *pal*-deficient *A. actinomycetemcomitans *mutant, D7SS-p, selectively changed. The susceptibility to erythromycin, but not to the β-lactams, increased. Previous studies have shown increased antibiotic susceptibility of strains with *tol-pal *gene mutations [59]. Additionally, mutations in genes encoding certain OMPs, such as *ssc *and *envA *of *E. coli *(encode 36- and 34-kDa OMPs, respectively), have resulted in hypersusceptibility to hydrophobic antibiotics, including erythromycin [60]. Our finding that the susceptibility of the *pal*-deficient *A. actinomycetemcomitans *mutant to the tested β-lactams did not increase may result from the inefficiency of the β-lactam antibiotics to kill slowly multiplying bacteria [61].

## Conclusion

Our results suggest that live *A. actinomycetemcomitans *cells release AaPAL in free-soluble form independent of vesicles. The proinflammatory and prochemotactic properties of AaPAL may contribute to producing a long-term immune challenge in persistent *A. actinomycetemcomitans*-associated infections. The extracellular release of PAL is also of more general interest because bacterial lipoproteins are increasingly being recognized as playing an important role in bacterial pathogenicity [7,14,62].

## Methods

### Bacteria and culture conditions

The test bacteria included 33 *A. actinomycetemcomitans *strains of 6 serotypes [63-65] and 15 genotypes [66,67]. *A. actinomycetemcomitans *strains were of oral (N = 31) and non-oral (N = 2) origin. Unless otherwise stated, strains were cultured on either supplemented blood agar plates [4], tryptic soy agar plates (TSA), or broth (TSB) (supplemented with 0.6% yeast extract, 0.8% glucose) (Difco, Sparks, MD, USA) and incubated in either CO_2_-enriched air (5%) or anaerobic atmosphere (85% N_2_, 10% H_2_, 5% CO_2_) at 37°C for 3 d. For biofilm growth [27], the bacteria were cultured in TSB in a 24-well cell culture plate (Nunc™, Nunc, Denmark) and incubated in either CO_2_-enriched air or anaerobic atmosphere, as detailed above, for 5 d. *V. cholerae *strains BML24/pHA 7 (*crp*^+^) and BML24/pBR322 (*crp*^-^) (Wai, unpublished) were cultured in Luria Bertani broth at 37°C overnight with shaking.

### Production of anti-AaPAL peptide antiserum

Antiserum was raised in rabbit against two synthetic peptides of AaPAL (N-terminal: 4–37 and C-terminal: 123–136), as described earlier [3].

### SDS-PAGE and immunoblot

Bacterial outer membrane fraction was extracted as previously described [4]. Bacteria suspended in PBS were used for whole cell protein preparation. SDS-PAGE and immunoblot were performed as previously described [4]. For immunoblots we used anti-AaPAL peptide antiserum [3], an antiserum against whole cells of *A. actinomycetemcomitans *serotype a [64], and *V. cholerae *cAMP receptor protein [68]. Unless specified otherwise, the antisera were used at final dilutions of 1:100 000, 1:1000, and 1:3000, respectively. Horseradish peroxidase-conjugated anti-rabbit secondary antibodies were used at a final dilution of 1:10000 or 1:20000. Immunoreactive bands were visualized using SuperSignal^® ^(Pierce, Rockford, IL, USA).

#### *pal*-deficient mutant strains

D7SS-p, the *pal*-deficient mutant derivative of strain D7SS (spontaneous smooth-colony variant of wild-type D7S), was constructed by gene replacement technique as described earlier [69]. The *pal*::Spe allele of D7SS-p was transferred to the wild-type fimbriated strain D7S, using natural transformation [70], generating D7S-p. The presence of the *pal*::Spe allele in D7S-p was confirmed by PCR using the primers PAL-Sm 5'-CTTTCCCGGGACATAACGG and PAL-Hd 5'-GCTGAAAGCTTATAGTG TAAGAG. The abolished AaPAL expression in the *pal*-deficient mutants was confirmed by immunoblot using anti-AaPAL peptide antiserum.

### PCR-restriction fragment length polymorphism (PCR-RFLP) of the *A. actinomycetemcomitans pal *sequence

For RFLP, PCR amplicons generated by the primers PAL-SA 5'-GCTGGTTCTGTGGCTGTGT and PAL-MK 5'-ACTGCACGACGGTTTTTAGC were purified (QIAquick^® ^Gel Extraction Kit, Qiagen GmbH, Helden, Germany) and digested with *Bsp*MI and *Dde*I (New England BioLabs, Beverly, MA, USA), respectively, prior to analysis on agarose (1.8%) gel electrophoresis.

### Phenotypic characterization of mutants

Phenotypic characteristics, such as colony size and density, cell morphology, growth rate, and antibiotic susceptibility were studied as follows:

#### Light microscopy

10-μl inocula containing 10^3 ^cells of strains D7SS, D7SS-p, D7S, and D7S-p were cultured on supplemented blood agar and TSA plates as described above and observed under a stereomicroscope (Nikon SMZ800, Tokyo, Japan). The cell morphology was evaluated using phase contrast microscopy at × 1000 magnification (Leica DMRBE).

#### Scanning electron microscopy (SEM)

The strains D7SS, D7SS-p, D7S, and D7S-p cultured on TSA plates were fixed (2.5% glutaraldehyde in PBS; RT, 5 min) and prepared according to established methods. The cells were dehydrated using increasing concentrations of ethanol, and thereafter dried from liquid CO_2 _by the critical-point drying technique [71] using a Polaron E-3000 CPD apparatus (Polaron Equipment Ltd, Watford, UK). The cover slips with attached bacteria were mounted on aluminum stubs with silver dag (Agar Scientific Ltd, Stansted, UK) and coated (approximately 15 nm of carbon and gold) in an Edwards E12 Vacuum Coating Unit (Edwards High Vacuum Ltd, Crawley, UK). Bacterial cell morphology was examined in a Cambridge S360ixp scanning electron microscope run with a LaB6 electron emittor (Leica Cambridge Ltd, Cambridge, UK) and the micrographs were recorded from randomly selected areas with standardized magnifications.

#### Growth rate

For determining the growth rates for the strains D7SS and D7SS-p, log-phase cultures were used to inoculate TSB. The growth was measured as turbidity at OD_600_.

#### Antibiotic susceptibility

The D7SS and D7SS-p strains were grown in lawn cultures (1 × 10^8 ^CFU/plate) on TSA plates on which discs of erythromycin (15 μg/disc), cefotaxime (30 μg/disc), and penicillin (10 U/disc) (BD Biosciences, San Jose, CA, USA) were placed. The plates were incubated at 37°C in CO_2_-enriched air for 3 d. Inhibition zones were measured in millimeters.

### Isolation of outer membrane vesicles

Outer membrane vesicles were isolated as described previously [40] with some modifications. *A. actinomycetemcomitans *strains D7SS and D7SS-p were cultured on supplemented blood agar plates for 3 days as described above. Bacteria were harvested and suspended into sterile PBS and centrifuged at 5000 × g (10 min, RT) to pellet the bacteria. The supernatant was filtered (0.22 μm; Millipore, Billerica, MA, USA) to remove any remaining intact bacterial cells. The filtrate was ultracentrifuged at 125000 × g (3 h, 4°C) using a 70 Ti rotor (Beckman Instruments Inc. Palo Alto, CA, USA). The obtained pellet was lyophilized and suspended in sterile PBS. The outer membrane vesicle preparations were examined by electron microscopy as described earlier [40]. Briefly, the vesicle samples were stained with 0.5% uranyl acetate and examined by a JEM 2000 ET electron micoscope (JEDL, Akishima, Tokyo, Japan) at 100 KV.

### Cell culture insert model for studying extracellular release of AaPAL in vitro

Cell culture insert experiments were carried out using exposure to serum (2 and 8 h) and culture in broth (24 h) as follows: *A. actinomycetemcomitans *cells (1 × 10^9 ^CFU/ml) were suspended in heat inactivated 50% fetal calf serum (FCS) (Invitrogen Gibco, Carlsbad, CA, USA) in PBS. The cell suspension was transferred into inserts (membrane pore size 0.02 μm), which were placed in cell culture wells (Nunc) containing FCS. The procedure for culturing in broth was similar to that for FCS, except that the wells contained TSB and the bacterial cells were suspended in TSB to a concentration of 1 × 10^8 ^CFU/ml. Inserts containing serum or broth without bacteria served as negative controls.

For detection of released AaPAL, following incubation at 37°C in CO_2_-enriched air, 100 μl samples, referred to as "filtrates" henceforth, were taken from serum (at 0, 2, and 8 h) and broth (at 0 and 24 h) from the wells outside the inserts. Samples from serum and broth filtrates, containing 10 and 30 μg protein, respectively, were subjected to immunoblot analysis. The amount of AaPAL released into serum filtrate was quantified by densitometry (Quantity One^® ^software, Bio-Rad, Hercules, CA, USA) using purified AaPAL (2, 4, 6, and 8 ng) as a reference. As the intensities of AaPAL bands were very similar in all three independent experiments, data from only one experiment was chosen for densitometric quantification.

To monitor the viability of *A. actinomycetemcomitans *cells in serum and broth during incubation, 10-μl samples of the bacterial suspensions were taken from the inserts at the same time points as the samples were taken from the filtrates for immunoblot. Since bacterial cells might have sedimented to the bottom during incubation, the bacterial suspensions inside the inserts were pipetted in and out repeatedly 4–5 times to make the suspension homogeneous before taking out the samples for culture and viable counts. Appropriate serial dilutions of the samples were plated on supplemented blood agar plates and incubated at 37°C in CO_2_-enriched air for 3 d. To exclude bacterial contamination of the serum or broth filtrates, 10-μl samples were also taken and cultured on supplemented blood agar plates. Limulus assay (Cape Cod, E. Falmouth, MA, USA) was used for the detection of LPS in the filtrates.

To ensure that the integrity of the insert membrane remained intact (i.e., no leakage of bacterial cells) and that AaPAL found in the filtrate was not due to cell lysis in the insert, immunoblot was used to analyze the presence of a cytoplasmic marker protein, cAMP receptor protein, in the filtrates, as described above for the detection of AaPAL. For concentration, the filtrates were precipitated using ice-cold trichloroacetic acid (final concentration 10%).

To deliberately cause release of cytoplasmic proteins, *A. actinomycetemcomitans *D7SS cells (2 × 10^9 ^cells/ml) were lysed using Lysing matrix E provided in the FastDNA ^® ^SPIN Kit for Soil (BIO 101, Q-Biogen, Montreal, Canada) with Mini-BeadBeater-8 (BioSpec Products Inc., Bartlesville, OK, USA) at highest speed for 3 min. The lysed cells were incubated in broth in the cell culture insert as described above. Unlysed cells were incubated similarly as a negative control. After 8 h incubation, filtrates collected from the wells were TCA-precipitated and the cAMP receptor protein was detected by immunoblotting as above.

To investigate whether outer membrane vesicles passed through the insert membrane filters in our experimental setup, serum filtrates were ultracentrifuged using the same procedure for sample preparation and electron microscopic examination as described above for the isolation of outer membrane vesicles.

### Cytokine induction from human whole blood by AaPAL

AaPAL was purified by affinity chromatography as described previously [3]. Each microgram of purified AaPAL had LPS contamination of less than 5 pg, an amount which was shown not to contribute to AaPAL-induced cytokine production [72]. AaPAL TranSignal human cytokine antibody arrays (Panomics, Redwood City, CA, USA) were used to profile cytokines produced by human whole blood. Blood sample from a healthy individual was drawn via venipuncture into heparin-containing tubes (BD Biosciences). Blood was immediately stimulated with AaPAL (final concentration 1 and 5 μg/ml blood; 37°C, 5% CO_2_, 8 h), followed by centrifugation (5000 × g, 5 min) and cytokine analysis of the supernatants (5-fold dilutions) as instructed by the array manufacturer. Blood stimulated with AaPAL storage buffer served as a reference. Aliquots of blood were taken at 0 h and 8 h and plated on supplemented blood agar plates for bacterial contamination check. The experiment was repeated once.

### Statistical analysis

Unless otherwise indicated all experiments were repeated thrice. Mann-Whitney U test was used to determine the significance of the differences between the parental strain and the isogenic mutant in antibiotic susceptibilities and in the LPS amounts released into serum or broth. A P-value of less than 0.05 was regarded as statistically significant.

## Authors' contributions

MK contributed to the study design, performed most laboratory work (developed the insert model, performed most bacterial cultures and outer membrane protein preparations, carried out antibiotic susceptibility tests, immunoblots, PCR-RFLP analyses, blood stimulations, cytokine assays, vesicle preparations, and microphotography), and contributed to SEM analyses, to all data analysis, interpretation, and reporting, as well as preparation of the manuscript.

RI participated in the study design, contributed to production of anti-AaPAL peptide antiserum and to AaPAL purification, PCR-RFLP and biofilm cultures and respective data analyses and interpretations.

KE participated in Limulus assays for determining LPS concentrations and respective data analyses and interpretations.

DZ helped in SDS-PAGE and immunoblot of whole cell protein preparations, bacterial cultures and growth curve experiments and respective data analyses.

JO constructed the D7S-p mutant and was involved in drafting the manuscript

SNW contributed to outer membrane vesicle preparation and electron microscopy, provided antibodies against cAMP receptor protein, and commented on the manuscript.

CC constructed the *pal*-deficient mutant D7SS-p and contributed to the design of genetic manipulations of *A. actinomycetemcomitans *needed for constructing the D7S-p, and commented on the manuscript

SA conceived of the study, contributed to insert model development and data analysis, interpretation, and reporting, and is the responsible author for study coordination and manuscript preparation.
